# Insulin-Like Growth Factor 2 mRNA-Binding Protein 3 Modulates Aggressiveness of Ewing Sarcoma by Regulating the CD164-CXCR4 Axis

**DOI:** 10.3389/fonc.2020.00994

**Published:** 2020-07-03

**Authors:** Caterina Mancarella, Giulia Caldoni, Irene Ribolsi, Alessandro Parra, Maria Cristina Manara, Arthur M. Mercurio, Andrea Morrione, Katia Scotlandi

**Affiliations:** ^1^Laboratory of Experimental Oncology, IRCCS Istituto Ortopedico Rizzoli, Bologna, Italy; ^2^Department of Biomedical and Neuromotor Sciences, University of Bologna, Bologna, Italy; ^3^Department of Molecular, Cell and Cancer Biology, University of Massachusetts Medical School, Worcester, MA, United States; ^4^Department of Biology, Center for Biotechnology, College of Science and Technology, Sbarro Institute for Cancer Research and Molecular Medicine, Temple University, Philadelphia, PA, United States; ^5^Department of Pathology, Anatomy and Cell Biology, Thomas Jefferson University, Philadelphia, PA, United States

**Keywords:** IGF2BP3, metastases, CD164, CXCR4, Ewing sarcoma

## Abstract

Ewing sarcoma (EWS) is the second most common bone and soft tissue-associated malignancy in children and young adults. It is driven by the fusion oncogene EWS/FLI1 and characterized by rapid growth and early metastasis. We have previously discovered that the mRNA binding protein IGF2BP3 constitutes an important biomarker for EWS as high expression of IGF2BP3 in primary tumors predicts poor prognosis of EWS patients. We additionally demonstrated that IGF2BP3 enhances anchorage-independent growth and migration of EWS cells suggesting that IGF2BP3 might work as molecular driver and predictor of EWS progression. The aim of this study was to further define the role of IGF2BP3 in EWS progression. We demonstrated that high *IGF2BP3* mRNA expression levels correlated with EWS metastasis and disease progression in well-characterized EWS tumor specimens. EWS tumors with high *IGF2BP3* levels were characterized by a specific gene signature enriched in chemokine-mediated signaling pathways. We also discovered that IGF2BP3 regulated the expression of CXCR4 through CD164. Significantly, CD164 and CXCR4 colocalized at the plasma membrane of EWS cells upon CXCL12 stimulation. We further demonstrated that IGF2BP3, CD164, and CXCR4 expression levels correlated in clinical samples and the IGF2BP3/CD164/CXCR4 signaling pathway promoted motility of EWS cells in response to CXCL12 and under hypoxia conditions. The data presented identified CD164 and CXCR4 as novel IGF2BP3 downstream functional effectors indicating that the IGF2BP3/CD164/CXCR4 oncogenic axis may work as critical modulator of EWS aggressiveness. In addition, IGF2BP3, CD164, and CXCR4 expression levels may constitute a novel biomarker panel predictive of EWS progression.

## Introduction

Ewing sarcoma (EWS) is a rare disease but it is still the second most common malignancy of bone and soft-tissues affecting pediatric age. It is characterized by a very aggressive behavior, high propensity for metastasis, specifically to bone and lung. Metastases occur in 30–40% of patients with localized disease, while 20–25% of patients present metastasis at diagnosis. The current standard treatment of EWS is a multimodal approach consisting of surgery and/or radiotherapy, and a multiagent chemotherapy, which confers a 5-years survival rate of 70% in patients with localized tumor. On the contrary, metastatic disease has a survival rate of 30%, independently of intensification of chemotherapeutic regimens (1). The identification of novel therapeutic strategies and reliable predictors of patient survival is therefore imperative to improve the outcome for metastatic patients.

While the genetic features of EWS are well-defined (2), as well as the contribution of the fusion gene *EWS-FLI1* to oncogenesis (3), the molecular mechanisms underlying EWS metastases are still poorly understood (4, 5).

EWS is characterized by one of the lowest mutation rates among all tumors (6–8) and this genomic stability is conserved in metastasis (9). On the contrary, epigenetic heterogeneity is prevalent in EWS, and even increased in the metastatic stage (10–12).

In general, EWS metastatic progression is regulated by multifactorial mechanisms, which include the dynamic activation of stress-adaptive or cellular plasticity pathways mediated by epigenetic or post-transcriptional mechanisms (5, 13–16). Previous reports have shown that EWS cells increase the expression of genes associated with metastasis, such as *CXCR4* or *HIF-1*α, through post-translational histone modifications or RNA binding proteins (RBPs) activity (15, 17, 18). The G protein-coupled receptor chemokine (C-X-C motif) receptor 4 (CXCR4), activated by its natural ligand CXCL12, promotes migration of EWS cells (13, 15).

RBPs, along with microRNAs and long non-coding RNAs, represent major post-transcriptional regulators of gene expression, due to their ability to bind RNA sequences and finely tune nuclear export, translation/degradation rate, and intracellular localization of their multiple transcript targets (19).

We have recently identified insulin-like growth factor 2 mRNA-binding protein 3 (IGF2BP3) as a major determinant of EWS aggressiveness (20, 21). IGF2BP3 has a critical role in modulating multiple mRNAs, thereby regulating tumor initiation and progression (22). Accordingly, IGF2BP3 has recently emerged as putative prognostic biomarker for several tumors, including leukemia, carcinomas, and sarcomas (23).

In this study, we initially discovered that IGF2BP3 is significantly upregulated in metastatic lesions of EWS patients as compared to primary tumors, prompting us to investigate the molecular contribution of this RBP to the migration and dissemination of EWS cells. We then identified for the first time an oncogenic axis consisting of IGF2BP3/CD164 and CXCR4, which confers migratory advantage to EWS cells, particularly under stress-adaptive conditions.

## Materials and Methods

### Clinical Specimens

This study included EWS specimens from primary localized tumors and EWS metastatic lesions. EWS diagnosis and treatment were performed at the IRCCS Istituto Ortopedico Rizzoli (Bologna, ITALY). For diagnosis, histological, immunohistochemical, and molecular features were considered (24). For therapy, patients underwent local treatment (surgery and/or radiation therapy) and systemic induction chemotherapy. All the patients included in this study were enrolled in previously approved prospective studies (25, 26). For those patients who underwent surgery, histologic response to chemotherapy was examined in accordance to Picci et al. (27). Clinical-pathological features of EWS patients, updated to 2018, are summarized in Table 1.

**Table 1:** Clinical-pathological features of primary localized EWS patients included in the study.

**Characteristics**		**qRT-PCR (*****N*** **=** **48)**		**Microarray (*****N*** **=** **29)**		**IHC (*****N*** **=** **50)**	
		**No**	**%**	**No**	**%**	**No**	**%**
Gender	*Female*	11	22.9	10	34.4	15	30
	*Male*	37	77.1	19	65.5	35	70
Age	* ≤ 14 years*	22	45.8	10	34.4	14	28
	*>14 years*	26	54.2	19	65.6	36	72
Location	*Extremity*	33	68.7	22	75.8	47	94
	*Central*	4	8.3	2	6.9	3	6
	*Pelvis*	11	23	5	17.2	0	0
Surgery	*YES*	38	79.2	20	68.9	46	92
	*NO*	10	20.8	9	31	4	8
Local Treatment	*RxT*	10	20.8	9	31.0	4	8
	*RxT+Surgery*	11	23	5	17.2	8	16
	*Surgery*	27	56.2	15	51.7	38	76
Response to chemotherapy^*^	*Good*	10	26.3	5	25	15	32.6
	*Poor*	28	73.7	15	75	31	67.4

* *Data available for 38 patients in qRT-PCR, for 20 patients in microarray and for 46 cases in IHC*.

*qRT-PCR, quantitative Real-Time PCR; IHC, immunohistochemistry, RxT, radiotherapy, EWS, Ewing sarcoma*.

### Cell Lines

For *in vitro* studies, the following patient-derived EWS cell lines were employed: A673 cells were provided by Dr. H. Kovar (St. Anna Kinderkrebsforschung, Vienna Austria) while TC-71 cells were provided by T.J. Triche (Children's Hospital, Los Angeles, CA). Cell lines authentication was executed by short tandem repeat (STR) polymerase chain reaction (PCR) analysis using a PowerPlex ESX Fast System kit (Promega, Madison, WI, USA) and the last control was performed in December 2017. Absence of mycoplasm contamination was assessed every 3 months using MycoAlert mycoplasma detection kit (Lonza, Basel, Switzerland). Stable silencing of IGF2BP3 was achieved using short hairpin RNA (shRNA; TRCN0000074673) included in a pLKO.1 vector, and subsequent selection in puromycin (2 μg/ml; Sigma, St. Louis, MO, USA), as previously described (20, 21). Cell lines were cultured as previously reported (28). For hypoxia studies, cells were cultured in 1% O_2_ using a Galaxy 14S incubator (New Brunswick, Eppendorf, Milano, ITALY) at 37°C and 5% CO_2_.

Transient silencing of CD164 was performed using short interfering RNA (siRNA) from GE Healthcare Dharmacon (Lafayette, CO, USA); SMART POOL siGENOME_siRNA (M-016196-00-0020). As control, siGENOME_non-targeting siRNA was employed (D-001206-13-05). siRNAs (80 nM) were transfected into EWS cells using TransIT-X2 (Mirus, Madison, WI, USA) in accordance with the manufacturers' protocol.

### RNA-seq and Bioinformatics Analyses

RNA extraction, cDNA libraries, sequencing, reads alignment, and normalization were performed as previously described (21). Hierarchical supervised clustering was performed using GeneSpring 11.02 software on differentially expressed genes using Pearson's correlation. Enrichment analysis of differentially expressed genes was performed using MetaCore software (GeneGo, Thomson Reuters).

### Gene Expression Analysis

Extraction of total RNA from snap-frozen tissue samples, human mesenchymal stem cell (hMSC) primary cultures, and EWS cell lines was carried out using TRIzol^TM^ Reagent (Invitrogen, Carlsbad, CA, USA). Quantity and quality of obtained RNA were measured by NanoDrop (NanoDrop ND1000, ThermoFisher Scientific, Waltham, MA, USA) and/or by electrophoresis analysis. Reverse transcription was performed using High Capacity cDNA Reverse transcription kit (Applied Biosystems, Foster City, CA, USA). Obtained cDNA was amplified by quantitative Real-Time PCR (qRT-PCR) in a ViiATM 7 Real-Time PCR System (Applied Biosystems). Predesigned TaqMan probe (Applied Biosystem) was employed for *IGF2BP3* (Hs00559907_g1) expression level measurement. Primers set for *CD164 (*Fw: 5′-GAGTGCTGTAGGATTAATTGGAAAAT-3′, Rv: 5′-GGGAGGAATGGAATTCTGC-3′), *CXCR4* (Fw:5′-ACGCCACCAACAGTCAGAG-3′, Rv: 5′-AGTCGGGAATAGTCAGCAG-3′), and *Nanog* (Fw: 5′-CCTATGCCTGTGATTTGTGG-3′, Rv: 5′-GATCCATGGAGGAAGGAAGA-3′) were employed for SYBR green quantization. Primer pairs for *GAPDH*, used as a reference gene, were employed as reported previously (29). RT^2^ Profiler Cancer Inflammation and Immunity Crosstalk PCR Array, profiling 84 genes involved in those pathways, was purchased from Qiagen (Hilden, Germany). Relative expression of analyzed transcripts was quantified following the 2^−ΔΔCt^ method (30).

### Immunohistochemistry

Paraffin-embedded EWS specimens were incorporated in tissue microarrays (TMAs) and processed for immunohistochemistry (IHC) using an avidin–biotin–peroxidase method (Vector Laboratories, Inc., Burlingame, CA, USA). An overnight incubation with the following primary antibodies was performed: anti-CD164 (sc-271179, Santa Cruz Biotechnology, Dallas, TX, USA) diluted 1:50, anti-CXCR4 (ab2074, Abcam, Cambridge, UK) diluted 1:50, anti-IGF2BP3 (sc-47893, Santa Cruz Biotechnology) diluted 1:50. Samples were classified as follows: negative, when no staining was observed; positive when weak, moderate, or strong staining was observed.

### Western Blotting

For western blotting analysis, cells were harvested, rinsed with PBS and lysed with ice-cold lysis buffer (50 mM TrisHCl pH = 7.4, 150 mM NaCl, 1% Nonidet P-40 (NP-40), 0.25% sodium deoxycholate, 1 mM EGTA, 1 mM sodium fluoride, protease, and phosphatase inhibitors). Western blotting was performed according to standard procedures. Membranes were incubated overnight with the following primary antibodies: anti-IGF2BP3 (RN009P, dilution 1:20000, MBL International, Woburn, MA, USA), anti-CXCR4 (ab124824, dilution 1:1000, Abcam), anti-CD164 (AF5790, dilution 1:1000, R&D Systems, Minneapolis, MN, USA), anti-HIF-1α (sc-10790, dilution 1:2000, Santa Cruz Biotechnology), and anti-GAPDH (sc-25778, dilution 1:10000, Santa Cruz Biotechnology). The following secondary antibodies were used: anti-rabbit (NA934) and anti-mouse (NA9310V, GE Healthcare, Little Chalfont, UK) or anti-sheep (HAF016, R&D Systems) antibodies conjugated to horseradish peroxidase.

### Motility Assay

Migration capability of EWS cells was established using Trans-well chambers (CoStar, Cambridge, MA, USA). 1 × 10^5^ cells diluted in IMDM plus 1% FBS were seeded in the upper compartment, whereas IMDM plus 1% FBS and CXCL12 (100 ng/ml, ab9798, Abcam) were placed in the lower compartment of the chamber. After an overnight incubation, under normoxia or hypoxia, migrated cells were fixed in methanol. Cells were subsequently stained with Giemsa and counted.

### Confocal Microscopy

Cells seeded on fibronectin-coated coverslips (Sigma) were serum starved for 24 h and pretreated with 80 μM dynasore (S8047, Selleckchem, Houston, TX, USA), or DMSO as control, in 1% FBS medium for 30 min at 37°C. Cells were then stimulated with CXCL12 (100 ng/ml, Abcam) in 1% FBS medium for 5 min at 37°C. Cells were fixed in 4% paraformaldehyde, permeabilized in Triton X-100 0.15%-PBS, blocked in 4% BSA and incubated with the following primary antibodies: anti-CXCR4 (ab124824, dilution 1:100, Abcam); anti-CD164 (sc-271179, dilution 1:50, Santa Cruz Biotechnologies). Anti-rabbit rhodamine (#31686, dilution 1:100, Thermo Scientific) and anti-mouse FITC (#31569, dilution 1:100, Thermo Scientific) were employed as secondary antibodies. Nuclei were counterstained with Hoechst 33256 (Sigma). Confocal analysis was performed using Nikon A1R confocal microscope with a Plan Apo 60x/NA 1.4 DIC N2 objective (Nikon, Minato, Tokyo, JP). To determine colocalization of the proteins of interest, Z-stacks were acquired at 0.25 μm intervals using the following settings: 1,024 × 1,024 pixel, 2 scanner zoom, 0.5 μm scan speed. Images were analyzed using Nis Elements AR4.20.01 software (Nikon, Minato, Tokyo, JP). Colocalization was quantified by Mander's Colocalization Coefficient as we previously performed (31).

### Ribo-Immunoprecipitation (RIP) Assay

The RiboCluster Profiler RIP-Assay kit (MBL International, Woburn, MA, USA) was used to identify IGF2BP3/transcript interactions, according to the manufacturers' protocol. For immunoprecipitation, anti-IGF2BP3 antibody (MBL International) or normal IgG (MBL International), used as a negative control, were used. Obtained RNA was reverse transcribed and qRT-PCR on equivalent amounts of cDNA was performed.

### Statistical Methods

Differences among means were tested using a one-way ANOVA, if more than two groups were present, or Student's *t*-test. Spearman's rank test was employed to establish correlation between continuous variables. Spearman's correlation coefficients (*r*) were defined as weak (0.1< *r* < 0.39), moderate (0.4< *r* < 0.69), or strong (0.7< *r* < 0.89), based on published definitions (32). Chi-square test was employed to establish correlation between categorized variables. Two-sided *p* < 0.05 was considered statistically significant.

## Results

### IGF2BP3 Is Associated With Metastasis Formation and With Chemokine Signaling

**Figure 1:**
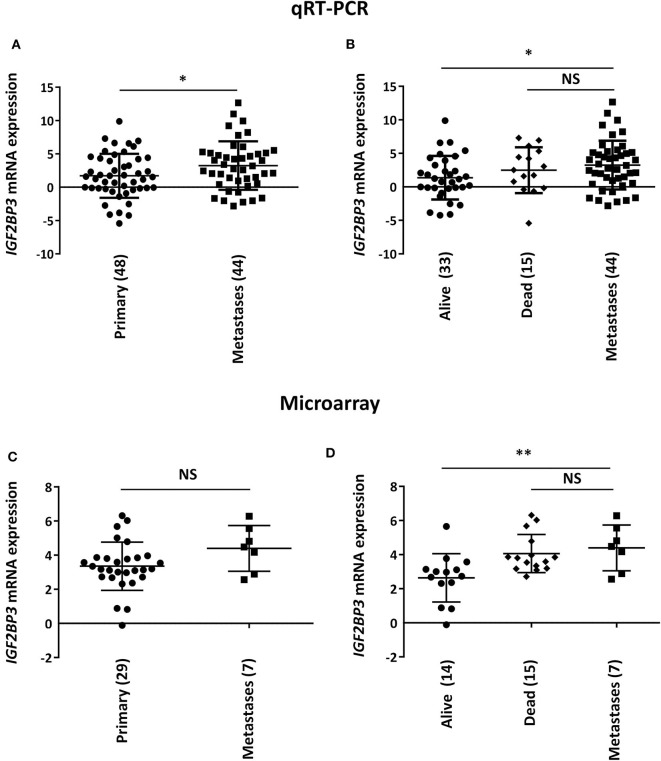
Correlation between *IGF2BP3* and metastatic disease in EWS patients. Scatter plot analysis of *IGF2BP3* mRNA levels determined via **(A,B)** qRT-PCR or **(C,D)** microarray (GSE12102) in primary or metastatic EWS lesions. Differential expression between the groups was determined using **(A,C)** Student's *t*-test or **(B,D)** one-way ANOVA with respect to metastases. Mean ± *SD* of relative mRNA expression reported as log_2_ is shown. Human mesenchymal stem cells were used as calibrator. Number of analyzed cases is reported below each plot. *p < 0.05; **p < 0.01. NS, not significant.

To initially explore a possible correlation between IGF2BP3 and EWS metastasis, we measured *IGF2BP3* mRNA expression levels in 44 metastatic EWS lesions using as controls 48 primary untreated tumors from patients with localized disease at diagnosis (Table 1). Metastatic specimens displayed significantly higher expression of *IGF2BP3* mRNA as evaluated by qPCR (Figure 1A).

Notably, when the subset of primary tumors was divided according to overall survival of patients (alive vs. dead from disease), we found that the significant difference of *IGF2BP3* mRNA expression levels was only maintained when compared to tumors derived from patients with favorable overall outcome (alive; Figure 1B). This observation was confirmed in a different set of tumors previously analyzed by microarray analysis (29 primary tumors vs. 7 metastasis; Table 1) (9, 21). We did not detect a significant difference in *IGF2BP3* expression levels between primary and metastatic tumors (Figure 1C). However, *IGF2BP3* expression levels were upregulated in metastatic lesions as in primary tumors of patients dead from disease but they were significantly lower in primary tumors of patients who did not experience any recurrence or were alive at 10 years from diagnosis (Figure 1D).

To further define whether IGF2BP3-regulated mechanisms might have clinical impact, we took advantage of another set of 14 tumors analyzed by RNAseq (21). We compared the genetic expression profile of three primary localized EWS cases with the highest expression of *IGF2BP3* to three primary localized EWS cases with the lowest, if any, expression of *IGF2BP3* and identified a signature of 814 differentially expressed genes (615 upregulated and 199 downregulated, *P* < 0.05; one-way ANOVA; Supplementary Table 1). This signature clearly separated the two groups with different *IGF2BP3* expression levels when hierarchical supervised clustering was performed (Supplementary Figure 1). Enrichment analysis using GeneGo annotation revealed the specific involvement of immunological and chemokine-mediated signaling pathways (Table 2).

**Table 2:** Enrichment analysis performed on 814 differentially expressed genes identified via RNAseq analyses in IGF2BP3-high vs. IGF2BP3-low expressers primary localized EWS cases using GeneGo annotation.

**#**	**Pathway maps**	**Total**	***p*-value**	**FDR**	**In data**	**Network objects from active data**
1	*Immune response_ Antigen presentation by MHC class I: cross-presentation*	99	4.4E−20	5.7E−17	30	IRAP, Rab-3B, Syk, Cathepsin L, HSP70, Dectin-1, Fc gamma RI, C1q, Fc epsilon RI gamma, IP-30, TIM-3, Adipophilin, SREC-I, Cathepsin S, MSR1, MANR, FCGR3A, Rab-35, DAP12, TLR4, Rab-32, OLR1, TLR7, CD74, Cathepsin B, TLR2, gp91-phox, p67-phox, VAMP8, Fc gamma RII alpha
2	*Chemokines in inflammation in adipose tissue and liver in obesity, type 2 diabetes and metabolic syndrome X*	48	1.3E−19	8.5E−17	22	ITGA4, ITGAX, ITGAM, ICAM1, IL-1 beta, CCL2, MIP-1-alpha, Fc gamma RI, PLAUR (uPAR), MANR, IL-8, FCGR3A, MHC class II, VCAM1, TLR4, CD86, CD68, **CXCR4**, CD163, CD45, TLR2, CD14
3	*Macrophage and dendritic cell phenotype shift in cancer*	100	8.6E−14	3.7E−11	24	ITGAM, Activin A, PGE2R2, c-Rel (NF-kB subunit), IL-1 beta, EPAS1, PGE2R4, ILT4, IDO1, DLL1, MSR1, MHC class II, WNT5A, M-CSF receptor, TLR4, TLR7, CD86, GM-CSF receptor, Gas6, ILT3, IRF5, TLR2, SHIP, CSF1
4	*Rheumatoid arthritis (general schema)*	50	7.6E−13	2.4E−10	17	IL-15, IL-18, ICAM1, MHC class II beta chain, IL-1 beta, Fc gamma RI, HLA-DRB, TNF-R2, HLA-DRB1, FCGR3A, MHC class II, VCAM1, TLR4, CD86, TLR2, CD4, CSF1
5	*Neutrophil chemotaxis in asthma*	38	1.4E−12	3.5E−10	15	C5aR, GRO-2, CCL2, MIP-1-alpha, HSP70, PI3K reg class IB (p101), IL-8, PTAFR, GRO-3, CCR1, G-protein alpha-i family, GRO-1, TLR2, PI3K cat class IB (p110-gamma), ENA-78
6	*Immune response_ Antigen presentation by MHC class II*	118	4.1E−12	8.1E−10	24	MHC class II alpha chain, Syk, Cathepsin L, MHC class II beta chain, Dectin-1, Fc gamma RII beta, Fc epsilon RI gamma, IP-30, HCLS1, Cathepsin S, MANR, HLA-DM, Cathepsin V, FCGR3A, MYO1E, MHC class II, TLR4, CLEC10A, OLR1, Legumain, CD74, TLR2, CD4, SWAP-70
7	*Basophil migration in asthma*	55	4.4E−12	8.1E−10	17	CCL18, ITGAM, C5aR, ICAM1, FPRL2, CCL2, MIP-1-alpha, PLAUR (uPAR), PI3K reg class IB (p101), IL-8, CCR1, G-protein alpha-i family, VCAM1, GM-CSF receptor, PLAU (UPA), PI3K cat class IB (p110-gamma), CCL13
8	*Immune response_Alternative complement pathway*	53	2.9E−11	4.6E−09	16	C5aR, C3a, C3, C5 convertase (C3b2Bb), Factor I, Factor Ba, C3b, CRIg, Factor Bb, C3aR, C3 convertase (C3bBb), iC3b, C3dg, Factor B, C3c, Clusterin
9	*Maturation and migration of dendritic cells in skin sensitization*	41	7.8E−11	1.1E−08	14	MHC class II alpha chain, ICAM1, MHC class II beta chain, IL-1 beta, MEKK1(MAP3K1), HLA-DRB, TNF-R2, HLA-DRB1, IL-8, HLA-DRB3, MHC class II, HLA-DRA1, CD86, HLA-DRB5
10	*Cell adhesion_Integrin inside-out signaling in neutrophils*	77	1.8E−10	2.3E−08	18	Syk, ICAM1, Fc gamma RI, Cytohesin1, PI3K reg class IB (p101), IL-8, PTAFR, Lyn, Btk, DAP12, G-protein alpha-i family, Hck, GRO-1, Slp76, PI3K cat class IB (p110-gamma), IP3 receptor, FYB1, PREL1

### IGF2BP3 Regulates the Expression of CXCR4 Through CD164

**Figure 2:**
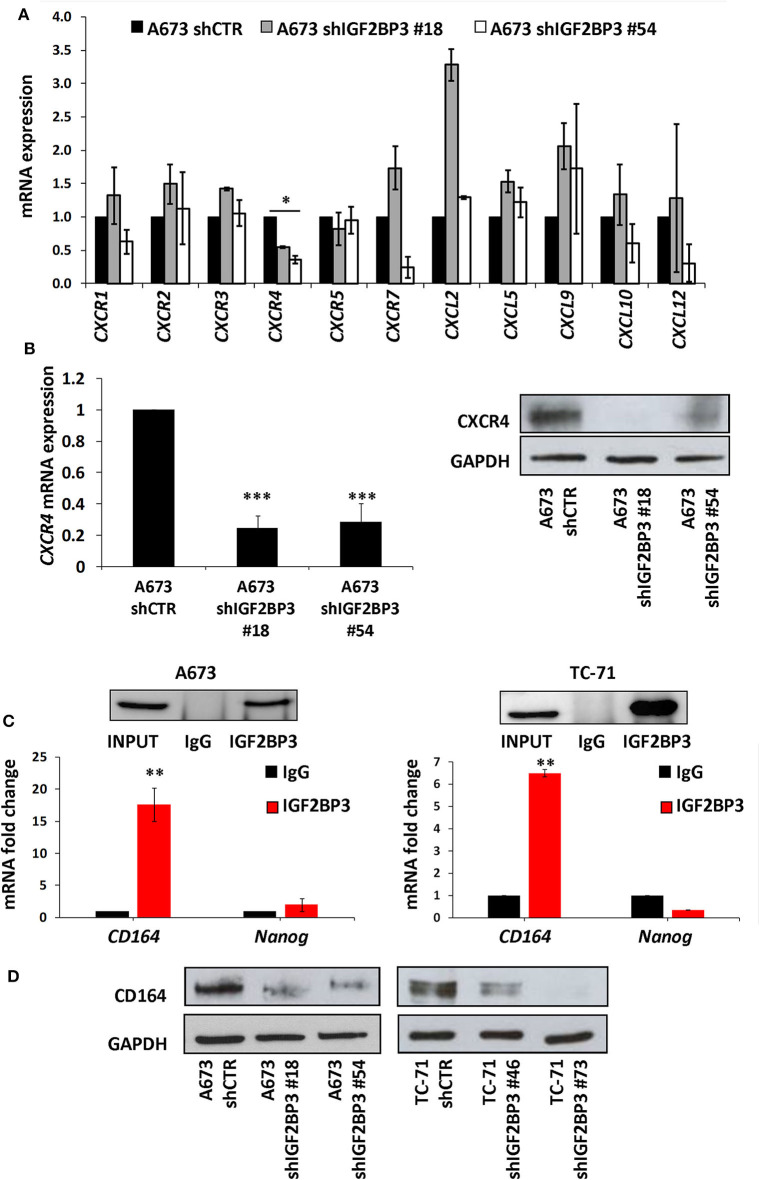
Characterization of the IGF2BP3/CD164/CXCR4 oncogenic axis in EWS cells. **(A)** qRT-PCR analysis performed with RT^2^ Profiler Cancer Inflammation and Immunity Crosstalk PCR Array on IGF2BP3-depleted or empty vector-transfected (shCTR) A673 EWS cells. Data are shown as 2^−ΔΔ*Ct*^ using A673 shCTR as calibrator and *GAPDH* as endogenous control. Mean ± *SE* of two independent experiments is shown. *p < 0.05, Student's *t*-test. **(B)** CXCR4 expression analyzed via (left) qRT-PCR or (right) western blot in IGF2BP3-depleted or empty vector-transfected (shCTR) A673 EWS cells. GAPDH was used as (left) housekeeping gene or (right) loading control. Histogram and western blot represent the sum of three independent experiments. ***p < 0.001, Student's *t*-test. **(C)** RIP assay performed on extracts from A673 and TC-71 EWS cells using an IGF2BP3 antibody or non-immune isotype matched IgG. *CD164* and *Nanog* mRNAs were quantified using qRT-PCR analysis. *Nanog* was used as a negative control. Western blot shows the specificity of IGF2BP3 antibody. Histograms represent mean ± *SE* of at least two independent experiments. **p < 0.01, Student's *t*-test. **(D)** Western blot depicting CD164 expression on IGF2BP3-depleted or empty vector-transfected (shCTR) A673 and TC-71 EWS cells. Representative western blots are shown. GAPDH was used as loading control.

To confirm the functional association between IGF2BP3 and chemokine signaling pathways, we took advantage of IGF2BP3-depleted experimental EWS cell models previously generated by shRNA approaches (21) (Supplementary Figure 2). We profiled control-transfected and IGF2BP3-depleted A673 EWS cells for genes encoding chemokine receptors and ligands using the RT^2^ Profiler Cancer Inflammation and Immunity Crosstalk PCR Array. Notably, only *CXCR4* was significantly downregulated in IGF2BP3-depleted A673 cells compared to controls (Figure 2A). Next, we confirmed by qRT-PCR and western blotting analyses downregulation of CXCR4 at mRNA and protein levels in IGF2BP3-silenced cells (Figure 2B), suggesting that CXCR4 might work as novel downstream effectors of IGF2BP3 action.

**Figure 3:**
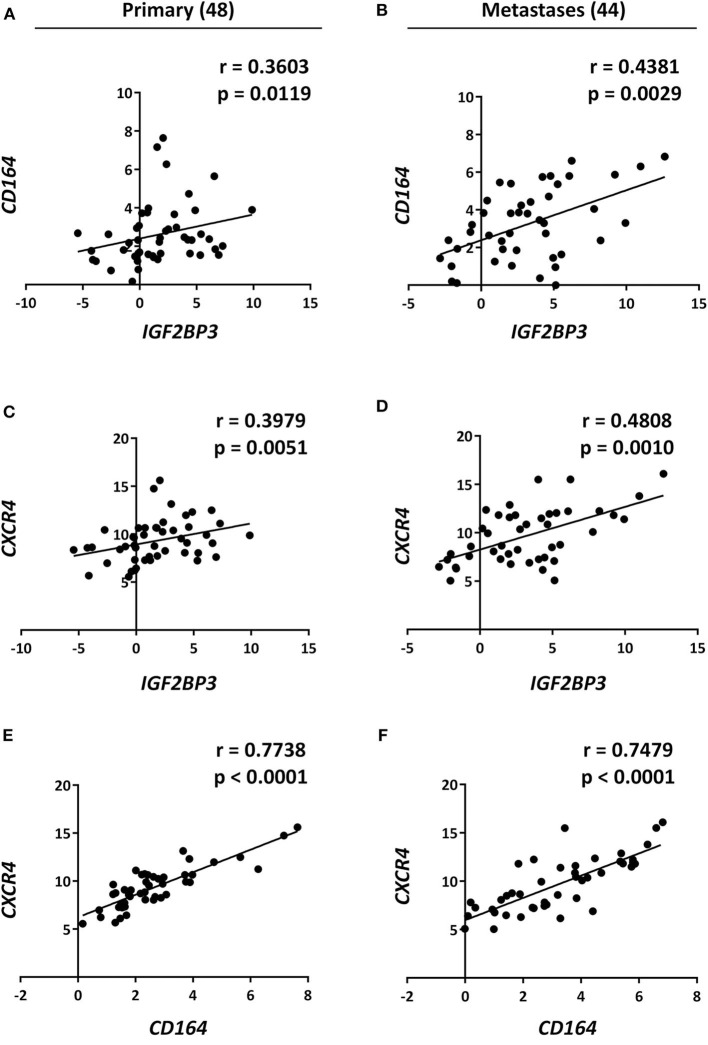
Analysis and correlation of *IGF2BP3, CD164*, and *CXCR4* mRNA levels in EWS patients. Scatter plots displaying correlations between *IGF2BP3, CD164*, and *CXCR4* mRNA levels in EWS **(A,C,E)** primary tumors and **(B,D,F)** metastatic lesions analyzed via qRT-PCR. Number of analyzed cases is reported above each column. Relative mRNA expression reported as log_2_ is shown. Human mesenchymal stem cells were used as calibrator. Correlation coefficient (*r*) and *p-*value were calculated using Spearman's rank test.

Data from the literature indicate that IGF2BP3 modulates the expression of CD164 (33, 34), a type 1 integral transmembrane sialomucin involved in the regulation of adhesion and migration of tumor cells (35, 36). Significantly, CD164 regulates CXCR4 function in different tumor types (36–38). Thus, we initially investigated a possible functional interaction between IGF2BP3 and *CD164* mRNA by RIP assay. In both A673 and TC-71 EWS cells *CD164* was significantly enriched in samples immunoprecipitated with anti-IGF2BP3 antibody as compared to IgG-immunoprecipitated control samples (Figure 2C). In addition, stable depletion of IGF2BP3 in A673 and TC-71 cells (Supplementary Figure 2) was associated with a significant reduction of CD164 protein expression levels as demonstrated by immunoblot analysis (Figure 2D). Next, we analyzed by qRT-PCR *IGF2BP3, CD164*, and *CXCR4* expression levels in clinical samples. We confirmed statistical association among the three molecules in both the 48 primary localized tumors and 44 metastatic lesions previously described (Figures 3A–F). Because Spearman coefficients (*r*) still indicated a weak to moderate correlation between *IGF2BP3* and *CD164* or *CXCR4* while a strong correlation between *CD164* and *CXCR4* (32), we further investigated the IGF2BP3/CD164/CXCR4 association by IHC in an independent cohort of 50 primary tumors (Table 1). The analyses confirmed a significant association at protein level between CD164 expression with both IGF2BP3 (*p* = 0.05, Chi-square test) and CXCR4 (*p* = 0.04, Chi-square test) (Table 3, Supplementary Figure 3).

**Table 3:** Association between CD164, CXCR4, and IGF2BP3 according to Chi-square test in 50 primary localized EWS cases analyzed by IHC.

**CD164**	**Negative**	**Positive**	***p*-value**
**CXCR4**			0.04
**Negative**	5	7	
**Positive**	5	30	
**IGF2BP3**			0.05
**Negative**	6	7	
**Positive**	7	30	

*CXCR4, not evaluable in three cases*.

Taken together these data support a role of IGF2BP3 in regulating the CD164/CXCR4 complex and demonstrate the evidence of an IGF3BP3-CD164-CXCR4 oncogenic axis critical for EWS progression.

### The IGF2BP3/CD164/CXCR4 Axis Affects Migration of EWS Cells in Response to CXCL12 and Under Hypoxia Conditions

**Figure 4:**
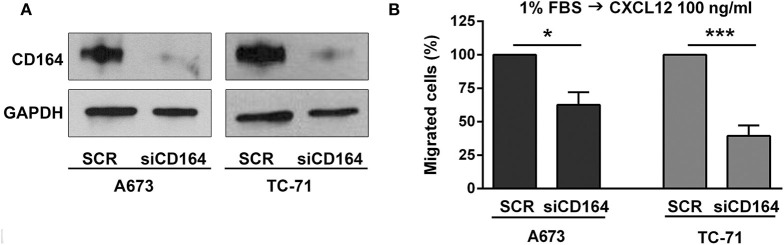
Functional relevance of CD164 in EWS cells. **(A)** CD164 silencing was achieved in A673 and TC-71 EWS cells after 72 h of transfection of siCD164 (80 nM) or scrambled control siRNA (SCR; 80 nM). GAPDH was used as the loading control. **(B)** Histogram shows the migration of A673 and TC-71 cells treated with siRNA or SCR using a CXCL12 (100 ng/ml) gradient. Mean ± *SE* of at least two independent experiments is shown. *p < 0.05; ***p < 0.001, Student's *t-*test.

While the role of CXCR4 in regulating migration of EWS cells has been previously established (13, 15), there are no data at the moment supporting the role of CD164 in modulating EWS cancer cells motility. Thus, we used siRNA approaches and transiently depleted CD164 in A673 and TC-71 cells. We obtained a robust CD164 depletion in both cell lines (Figure 4A), which determined a significant inhibition of EWS cell motility in condition of chemotactic stimulus toward a CXCL12 gradient (Figure 4B), supporting the notion that CD164 might act as an adjuvant factor of CXCR4 signaling in EWS cells.

**Figure 5:**
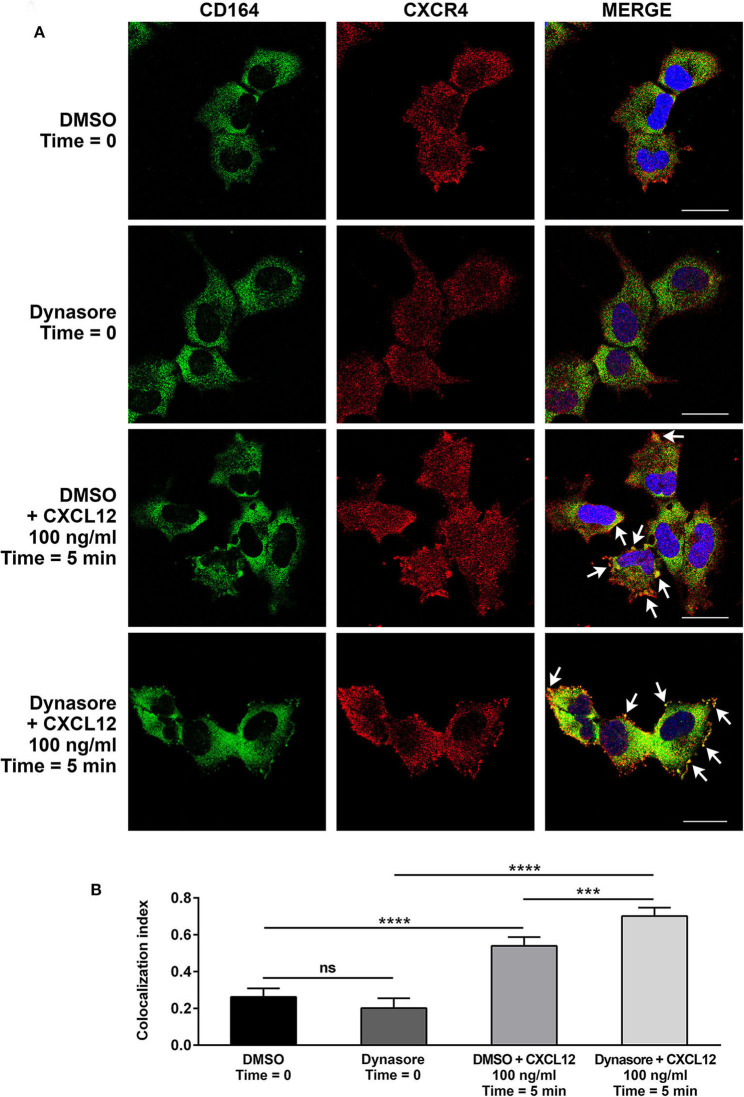
CD164 and CXCR4 colocalize upon CXCL12 stimulation in EWS cells. **(A)** Colocalization of CD164 and CXCR4 was assessed in A673 cells by immunostaining and confocal microscopy. Cells were pretreated with dynasore (80 μM), or DMSO as control, and then stimulated with CXCL12 100 ng/ml for 5 min (Time = 5 min), or left unstimulated (Time = 0). Images were taken using confocal microscopy and representative single *Z*-stack pictures are shown (scale bar 25 μm). **(B)** *Z*-stacks were analyzed for colocalization by NIS Elements AR4.20.01 software (Nikon). Colocalization index is represented by histograms. Mean ± *SE* of an average of 30 independent fields is shown. ns, not significant; ***p < 0.01; ****p < 0.0001, one-way ANOVA.

We then investigated by confocal microscopy whether CD164 and CXCR4 might colocalize in A673 cell line. In CXCL12-unstimulated cells, a homogeneous distribution of CD164 and CXCR4 was observed in the cytoplasm and at the plasma membrane (Figures 5A,B). On the contrary, upon CXCL12 stimulation, CD164 and CXCR4 colocalized at the plasma membrane. To confirm that CD164 and CXCR4 indeed interacts at the plasma membrane, we repeated colocalization experiments supplementing CXCL12 with the general endocytosis inhibitor dynasore, a GTPase inhibitor that blocks dynamin activity, thus affecting both clathrin-dependent and -independent endocytic pathways (39). The combination of CXCL12 and dynasore enhanced colocalization of CD164 and CXCR4 (Figures 5A,B), confirming that this interaction likely occurs at the plasma membrane of A673 cells (Figure 5A, white arrows). Collectively these results suggest that CD164 and CXCR4 colocalize at the plasma membrane of A673 cells in CXCL12-dependent fashion.

**Figure 6:**
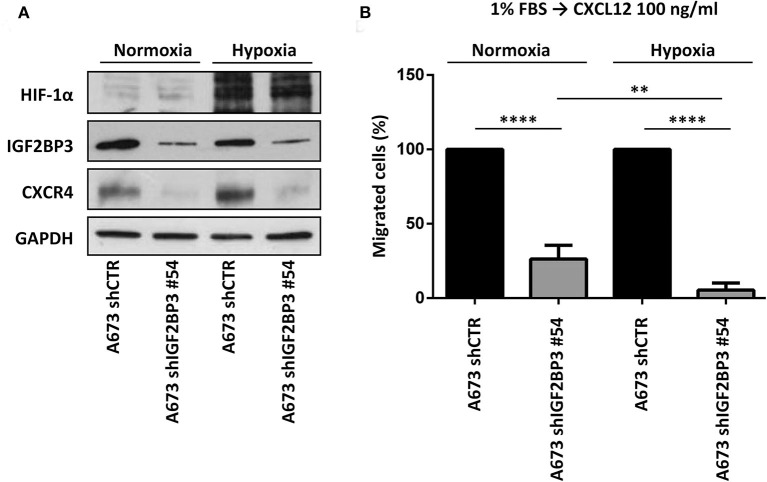
Functional relevance of IGF2BP3/CXCR4 axis in EWS cells. **(A)** Western blotting displaying HIF-1α, IGF2BP3, and CXCR4 expression in IGF2BP3-depleted or empty vector-transfected (shCTR) A673 EWS cells grown for 72 h under normoxia (21% O_2_) or hypoxia (1% O_2_). The western blots represent the sum of three independent experiments. GAPDH was used as the loading control. **(B)** Migration of IGF2BP3-depleted or empty vector-transfected (shCTR) A673 EWS cells using a CXCL12 (100 ng/ml) gradient under normoxia (21% O2) or hypoxia (1% O2). Mean ± *SE* of three independent experiments is shown. **p < 0.01; ****p < 0.0001, one-way ANOVA.

Because CXCR4 is induced in EWS cells exposed to hypoxia (13), a common condition of human tumor microenvironment (40), we investigated the contribution of the IGF2BP3/CD164/CXCR4 axis on CXCL12-evoked biological responses of EWS cells under normoxic (21% O_2_) or hypoxic conditions (1% O_2_). In line with previous evidence (13), EWS cells exposed to hypoxia showed induced expression of CXCR4 and of the hypoxia inducible factor alpha (HIF-1α), used as control (Figure 6A). Interestingly, IGF2BP3-silenced cells did not show CXCR4 expression, which was not increased under hypoxic conditions (Figure 6A). From the functional standpoint, the inhibitory effect on cell migration associated with IGF2BP3 depletion was amplified under hypoxic conditions. In fact, A673 cells silenced for IGF2BP3 showed reduced migration in response to CXCL12 either in normoxic or under hypoxia conditions (Figure 6B). Of note, the reduction was more evident in hypoxia condition (*p* = 0.005, one-way ANOVA), indicating that the impact of reduced expression of the IGF2BP3/CD164/CXCR4 oncogenic pathway may be stronger in the tumor microenvironment compared to physiological conditions.

## Discussion

EWS is characterized by a very low somatic mutational load (6–8) and high levels of inter- and intratumor epigenetic heterogeneity (10–12). Analysis of DNA methylation has highlighted a large spectrum of alterations, which reflect disease heterogeneity in term of stem cell differentiation and clinical outcome, and preclude the possibility of identifying subset of patients with differential risk of progression (12). Treatment of EWS is still based on high dense chemotherapy, with relevant impact on quality of life of survivors, who may be overtreated, and on outcome of high-risk patients, who should be considered for alternative drug regimens.

We have recently demonstrated that the mRNA binding protein IGF2BP3 constitutes an important biomarker for EWS (20, 21) as in fact high expression of IGF2BP3 in primary tumors is associated with poor prognosis of EWS patients (21). In addition, we demonstrated that IGF2BP3 increases anchorage-independent growth and migration of EWS cells (21) suggesting a putative role for IGF2BP3 as molecular driver of EWS progression. In this study, we demonstrated that: (A) High *IGF2BP3* mRNA expression levels correlate with EWS metastasis. (B) EWS tumors with high *IGF2BP3* mRNA expression levels are characterized by a specific gene signature enriched in chemokine-mediated signaling pathways. (C) IGF2BP3 regulates the expression of CXCR4 through CD164. (D) CD164 and CXCR4 colocalize at the plasma membrane of EWS cells upon CXCL12 stimulation. (E) IGF2BP3, CD164, and CXCR4 expression levels correlate in clinical samples. (F) The IGF2BP3/CD164/CXCR4 oncogenic axis promotes motility of EWS cells in response to CXCL12 and under hypoxia conditions.

Previously published data from our laboratory indicates that IGF2BP3 may exert its oncogenic action in EWS in both IGFs-dependent and -independent manner. IGF2BP3 loss promoted IGF1R downregulation and inhibited IGF1-evoked biological responses, thereby reducing cell growth and motility of EWS cells (20). IGF1R loss was associated with a compensatory mechanism driven by activation of the insulin receptor isoform A (IR-A) and its cognate ligand IGF2, which conferred enhanced sensitivity to dual IGF1R/IR inhibitors (20). On the other hand, IGF2BP3 expression is predictive of poor prognosis of EWS and regulate EWS aggressiveness independently of IGF1R action (21). The data presented here support the novel observation that in EWS cells IGF2BP3 might be a critical factor in regulating a specific cytokine pathway consisting of CD164 and CXCR4 signaling.

A role for CXCR4 in EWS has been previously demonstrated (13, 15, 41). Expression of CXCR4 is highly dynamic in EWS, and can be transiently induced by exposure to microenvironmental stress, like starvation, growth constraint and hypoxia (13). EWS cells characterized by high CXCR4 expression levels show increased invasion and migration capability, partially mediated by the intracellular activation of the Rho-GTPases, Rac1, and Cdc42 (13). Significantly, targeting the CXCL12/CXCR4 axis inhibited the aggressive phenotype, thereby indicating a potential contribution of CXCR4 signaling to EWS metastasis (13). In addition, in the model presented by Krook et al. stress induces the conversion of CXCR4-negative EWS cells to CXCR4-positive cells, thereby supporting the role of the CXCL12/CXCR4 signaling pathway in tumor progression (15). This switch is mediated, at least in part, by epigenetic modifications of the *CXCR4* promoter, which transitions from an inactive bivalent state to a univalent active state (15).

The adhesion receptor CD164 (endolyn), belonging to the sialomucin family, regulates the adhesion of CD34^+^ cells to bone marrow stroma, and the recruitment of those cells into cycle (37). CD164 associates with CXCR4 and cooperates with it in promoting CXCL12-mediated cell migration (37). CD164 depletion significantly attenuated the PI3K pathway but it did not alter MAPK activation, suggesting pathway specificity of CD164 action (37). A tumorigenic role of CD164 has been demonstrated in ovarian cancer where CD164 is upregulated in malignant ovarian cancer cell lines (38). CD164 overexpression in human ovarian epithelial surface cells increased CXCL12/CXCR4 expression, enhanced cellular proliferation, and colony formation, and suppressed apoptosis (38). Clinicopathological correlation analysis additionally indicated that CD164 upregulation was significantly associated with tumor grade and metastasis. In EWS, a putative role for CD164 in EWS transformation was suggested by Grunewald et al. who demonstrated that the thyroid receptor interacting protein 6 (TRIP6), belonging to the Zyxin family of proteins, is overexpressed in EWS and promotes cell growth, invasion, and migration through a transcriptional pro-invasive gene signature, which included *CD164* (42). However, CD164 mechanisms of action in EWS cells were not further characterized and its impact on tumor progression has never been evaluated.

According to previous evidences (33, 34), our data confirm a direct functional interaction between IGF2BP3 and CD164. In fact, IGF2BP3 and *CD164* are part of a complex detected by RIP assays, suggesting that IGF2BP3 might regulate mRNA stability and therefore expression levels of CD164. In turn, CD164 functionally interacts with CXCR4, thus regulating CXCR4 activation and CXCL12-dependent motility of EWS cells. In ovarian cancer cells, CD164 was localized in the cytosol and nucleus suggesting that nuclear CD164 might regulate CXCR4 promoter activity (38). The definition of downstream mechanisms of action of this signaling axis in EWS cells deserves further studies. It is important to mention that, in addition to IGF2BP3, additional proteins may contribute to CD164/CXCR4 regulation at post-transcriptional or epigenetic level, as suggested by the moderate associations between these 3 molecules observed in EWS cases. For instance, CXCR4 is regulated by dynamic post-translational histone modifications (15) while CD164 is a direct target of miRNA124, whose role in EWS has been previously reported (43, 44). Here, we put emphasis on the definition of an axis that may favor metastasis formation, the critical medical issue in the cure of EWS patients, and we provide evidence that support the possible use of drugs targeting IGF2BP3 and/or CXCR4 in high-risk patients with high expression of IGF2BP3/CD164/CXCR4 molecules. As recently reported, CXCL12 favors a pro-metastatic bone marrow niche in multiple myeloma, as well as in solid tumors with propensity to give bone metastases, including gastric, medullary thyroid, lung, prostate, and renal carcinomas (45). CXCR4-blocking agents, such as the neutralizing antibody MDX1338 or Ulocuplumab, were reported to efficiently reduce migration and invasion of osteosarcoma, alveolar rhabdomyosarcoma and myeloma cells and suppress the CXCR4-driven Epithelial-to-mesenchymal (EMT)-like phenotype (45–47), supporting the specific targeting of CXCR4 in therapy. More recently, the combination of MDX1338 and activated and expanded natural killer (NKAE) cell therapy was proposed as novel therapeutic approach to efficiently inhibit metastasis in mice (48). However, considering that CXCR4 may be up-regulated by epigenetic alterations or hypoxia-driven signaling which allow tumor cells to adapt and win the selection leading to tumor cell dissemination and metastasis in a new host environment, inhibition of IGF2BP3 may be more relevant. We have recently reported that inhibitors of Bromodomain and Extraterminal domain (BET) proteins can reduce expression of IGF2BP3 in EWS cells and synergize with vincristine (21). Further studies are necessary to develop more specific agents against this oncogenetic RBP.

In summary, the data presented in this work identified CD164 and CXCR4 as novel IGF2BP3 downstream functional effectors supporting the notion that the IGF2BP3/CD164/CXCR4 oncogenic axis may work as critical modulator of EWS aggressiveness. In addition, IGF2BP3, CD164, and CXCR4 expression levels may work as novel biomarkers predictive of EWS progression. Targeting of this axis may effectively prevent EWS disease dissemination.

## Data Availability Statement

The RNA-seq data discussed in this publication have been deposited in NCBI's Gene Expression Omnibus (49) and are accessible through GEO Series accession number GSE150722. Microarray data are accessible through GEO Series accession number GSE12102 (9).

## Ethics Statement

The studies involving human participants were approved by Ethical Committee of the IRCCS Istituto Ortopedico Rizzoli, Bologna, Italy (0019012/2016, 0005175/2018, and 0006158/2020). Written informed consent to donate material to the IRCCS Istituto Ortopedico Rizzoli tissue biobank for research purposes was obtained.

## Author Contributions

CM and KS: conception and design of the study. CM, GC, MM, IR, and AP: acquisition of data. CM, GC, MM, AMM, AM, and KS: analysis and interpretation of data. CM, AM, and KS: drafting or revising the work. All authors read and approved the final manuscript.

## Conflict of Interest

The authors declare that the research was conducted in the absence of any commercial or financial relationships that could be construed as a potential conflict of interest.

## Supplementary Material

Supplementary Table 1 List of differentially expressed genes identified via RNAseq analyses in IGF2BP3-high vs IGF2BP3-low expressers primary localized EWS cases.

Supplementary Figure 1 IGF2BP3 signature in EWS.

Supplementary Figure 2 Evaluation of IGF2BP3 silencing in EWS cellular models.

Supplementary Figure 3 IGF2BP3/CD164/CXCR4 association in EWS.
