# Supplementary figures and images for: Insulin-Like Growth Factor 2 mRNA-Binding Protein 3 Modulates Aggressiveness of Ewing Sarcoma by Regulating the CD164-CXCR4 Axis
Source: Front Oncol. 2020 Jul 3;10:994. doi: 10.3389/fonc.2020.00994 (PMC7347992; doi:10.3389/fonc.2020.00994)

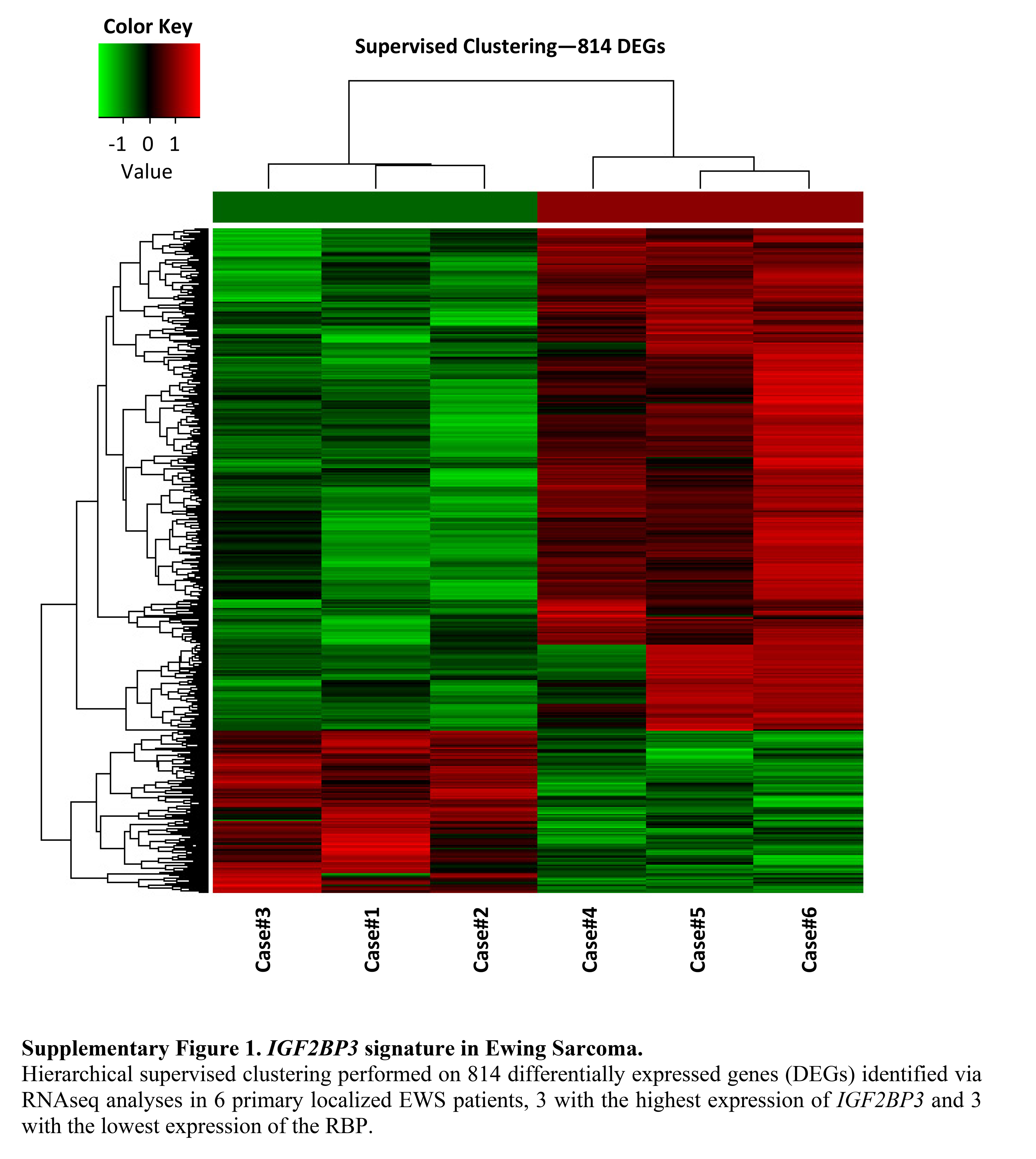

Supplement: Supplementary Figure 1 — IGF2BP3 signature in EWS. [file Image_1.TIF]

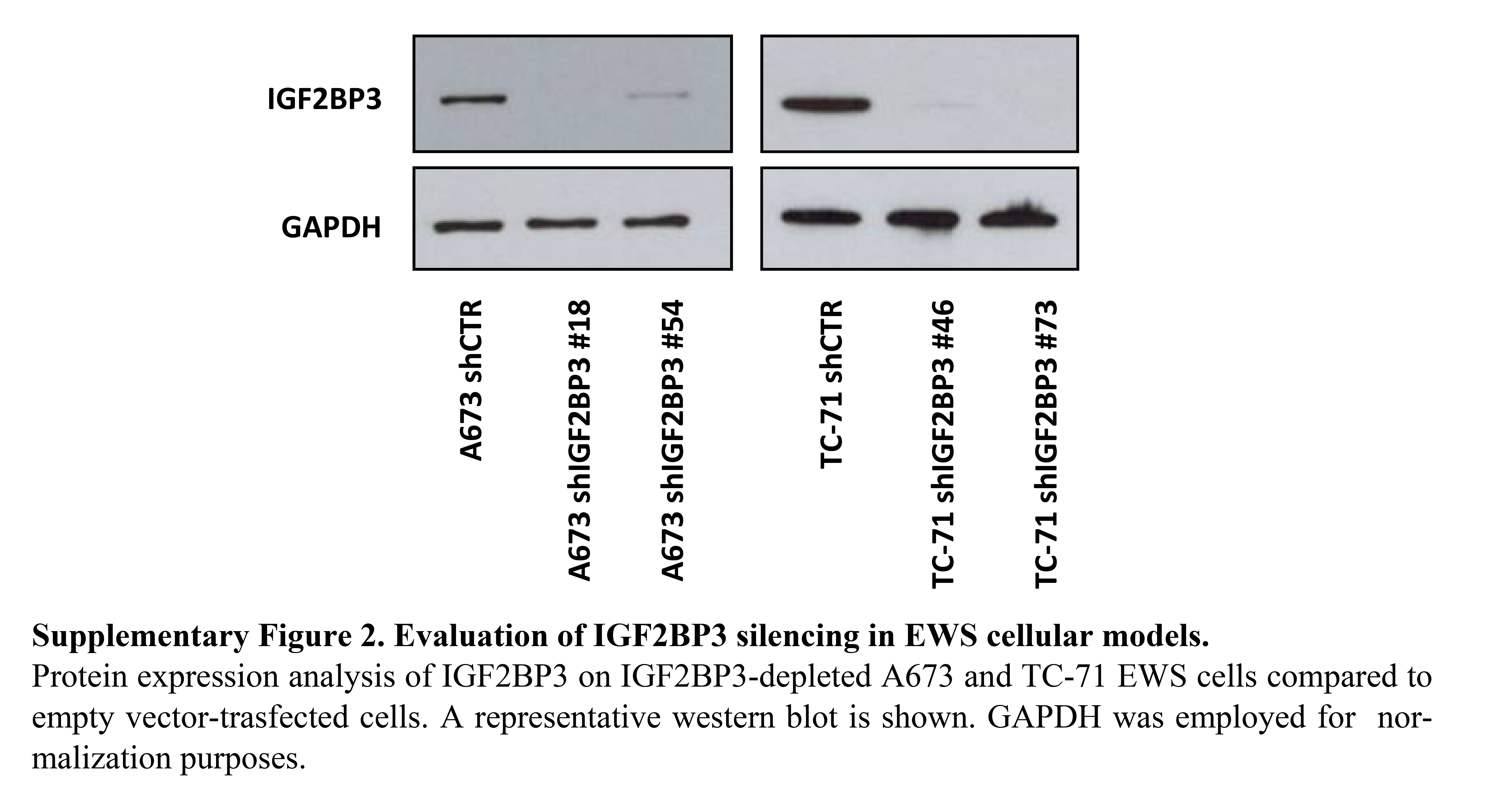

Supplement: Supplementary Figure 2 — Evaluation of IGF2BP3 silencing in EWS cellular models. [file Image_2.TIF]

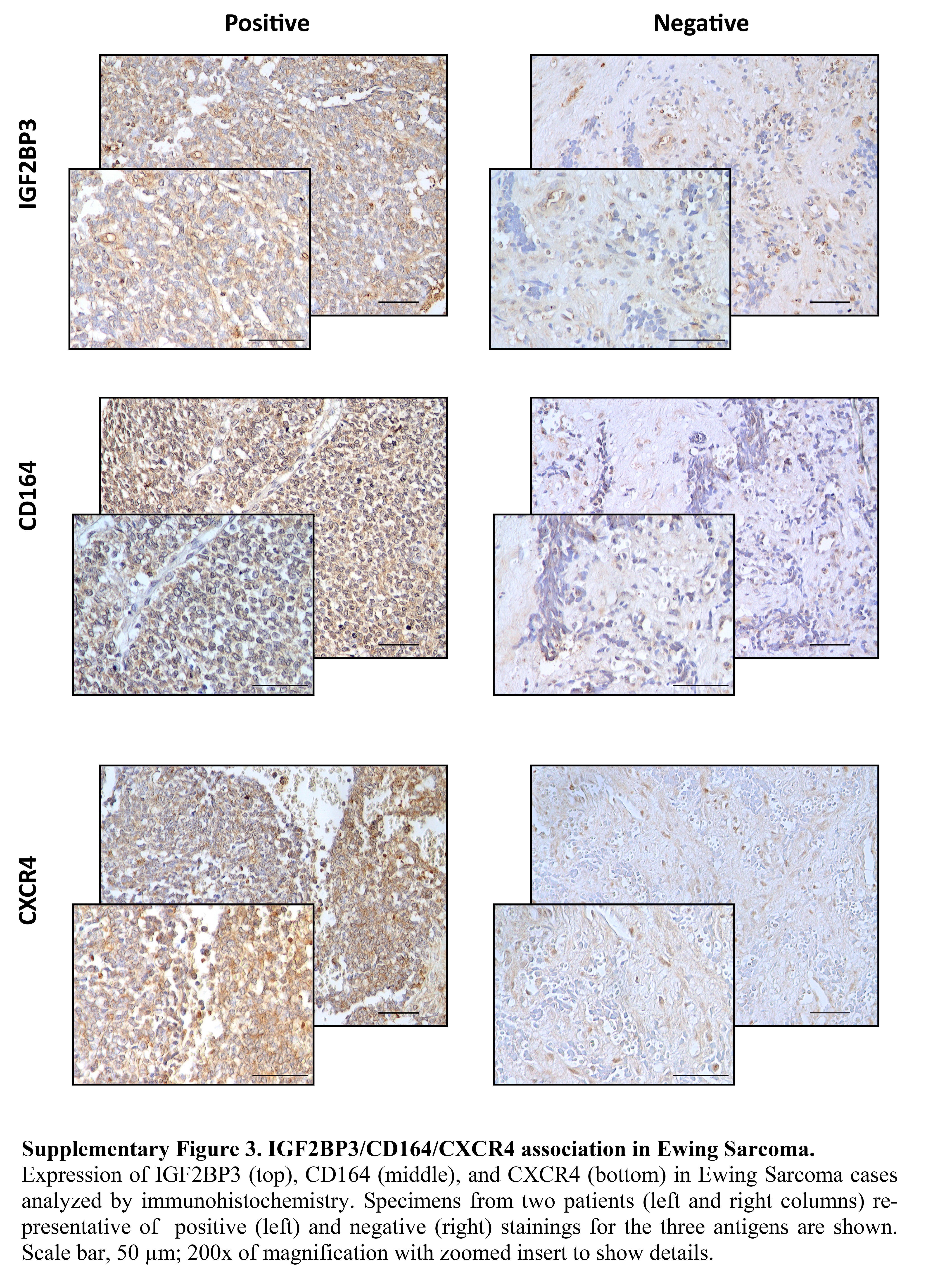

Supplement: Supplementary Figure 3 — IGF2BP3/CD164/CXCR4 association in EWS. [file Image_3.TIF]
